# Effects of aging and long‐term physical activity on mitochondrial physiology and redox state of the cortex and cerebellum of female rats

**DOI:** 10.14814/phy2.15542

**Published:** 2022-12-21

**Authors:** Paulo H. C. Mesquita, Shelby C. Osburn, Joshua S. Godwin, Michael D. Roberts, Andreas N. Kavazis

**Affiliations:** ^1^ School of Kinesiology Auburn University Auburn Alabama USA; ^2^ Edward Via College of Osteopathic Medicine Auburn Alabama USA

**Keywords:** antioxidants, brain, exercise, mitochondrial dynamics, mitochondrial function, oxidative damage

## Abstract

We investigated the effects of aging and long‐term physical activity on markers of mitochondrial function and dynamics in the cortex and cerebellum of female rats. Additionally, we interrogated markers of oxidative damage and antioxidants. Thirty‐four female Lewis rats were separated into three groups. A young group (YNG, *n* = 10) was euthanized at 6 months of age. Two other groups were aged to 15 months and included a physical activity group (MA‐PA, *n* = 12) and a sedentary group (MA‐SED, *n* = 12). There were no age effects for any of the variables investigated, except for SOD2 protein levels in the cortex (+6.5%, *p* = 0.012). Long‐term physical activity increased mitochondrial complex IV activity in the cortex compared to YNG (+85%, *p* = 0.016) and MA‐SED (+82%, *p* = 0.023) and decreased carbonyl levels in the cortex compared to YNG (−12.49%, *p* = 0.034). Our results suggest that the mitochondrial network and redox state of the brain of females may be more resilient to the aging process than initially thought. Further, voluntary wheel running had minimal beneficial effects on brain markers of oxidative damage and mitochondrial physiology.

## Graphical abstract

There were minimal changes in several markers of mitochondrial content, function, and dynamics in the cortex and cerebellum of female rats in response to both aging and long‐term physical activity. The brain mitochondrial physiology and redox homeostasis of females may be more resilient to the aging process than initially thought.

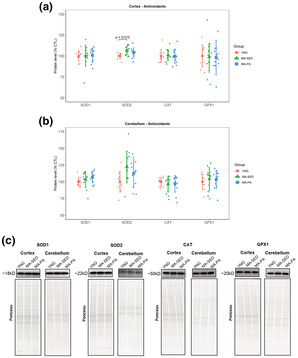

## 1 INTRODUCTION

Aging is characterized by a progressive decline in physiological function that is observed in several tissues, including the brain (Mattson & Arumugam, 2018). The impairment of mitochondrial function has been considered one of the major players in brain aging and has been linked to the development of many neurodegenerative diseases (Grimm & Eckert, 2017; Mattson & Arumugam, 2018). Indeed, age‐related decreases in different measures of mitochondrial function in the brain, including basal respiration (Lores‐Arnaiz et al., 2016), state 3 respiration (Lores‐Arnaiz & Bustamante, 2011; Thomsen et al., 2018) and the activity of the electron transport system complexes (I‐III, IV, and V) (Long et al., 2009; Navarro et al., 2002, 2004), have been repeatedly reported in the literature. In addition, disrupted mitochondrial dynamics has been recognized as a critical factor in the aging process and neurodegeneration (Marques‐Aleixo et al., 2012). Substantial sex disparities also exist with regards to age‐related metabolic changes in the brain (Grimm & Eckert, 2017; Zhao et al., 2016). Females have been shown to undergo changes in brain mitochondrial physiology, such as decreased expression of genes related oxidative phosphorylation, mitochondrial biogenesis and dynamics, at earlier stages than males (Zhao et al., 2016), and to be disproportionately affected by neurodegenerative diseases, such as Alzheimer's disease (Zhu et al., 2021).

Another important aspect of brain aging and mitochondrial dysfunction is a disruption of the balance between reduction–oxidation reactions (redox state). An increase in the production of reactive oxygen species (ROS) and a concomitant decrease in the antioxidant defense system lead to a state of chronic oxidative stress, causing damage to protein, lipids, and DNA (Sas et al., 2018). Although ROS are produced as a result of different enzymatic reactions, mitochondria are believed to be the main source of ROS (Grimm & Eckert, 2017), and are especially susceptible to oxidative damage. Therefore, mitochondrial dysfunction can lead to a vicious cycle of increased ROS production and damage to the organelles.

Physical exercise has been regarded as a therapeutic approach to improve mitochondrial function and the redox state of the brain, possibly preventing or delaying the development of neurodegenerative diseases (Marques‐Aleixo et al., 2012). For example, Marques‐Aleixo et al. (2015) reported that 12 weeks of exercise favorably altered different mitochondrial biomarkers in the cortex and cerebellum of young male rats. However, studies investigating the effects of both aging and long‐term physical activity on mitochondrial physiology, especially with regards to mitochondrial dynamics, and on the redox state of the brain of females are scarce. Therefore, the purpose of this study was to investigate the effects of aging and long‐term voluntary physical activity on markers of mitochondrial function and remodeling, oxidative damage, and the antioxidant defense system in the cerebral cortex and cerebellum of female rats.

## 2 MATERIALS AND METHODS

### 2.1 Animals

Female Lewis rats were obtained from a commercial vendor (Envigo) at 3 months of age. Rats were caged individually and maintained under controlled conditions with water and food ad libitum until 6 months of age was reached, when they were separated into three groups: (1) control group (YNG, *n* = 10); (2) sedentary (MA‐SED, *n* = 12); and physical activity (MA‐PA, *n* = 12). The YNG group was euthanized at 6 months and used as young reference group, while MA‐SED and MA‐PA groups were aged to 15 months. The MA‐PA group had 24‐h access to a running wheel and were allowed to run voluntarily for 9 months. The average weekly run distance and total run distance were recorded for each rat in the MA‐PA group. Running wheels of the exercise group were locked 20 h before euthanasia to avoid interference of acute effects of exercise. All experimental procedures were approved by the local Animal Care and Use Committee (protocol #: 2020–3647).

### 2.2 Animal euthanasia and tissue collection

Rats were euthanized by CO_2_ gas followed by cervical dislocation after a minimum of a 4 h‐fasting. After decapitation, brain cortex and cerebellum were rapidly removed, flash‐frozen in liquid nitrogen, and stored at −80°C for later molecular analyses.

### 2.3 Western blotting

Approximately 25 mg of each brain cortex and cerebellum were homogenized in cell lysis buffer (Cell Signaling) using tight‐fitting plastic pestles. Protein concentrations were determined using a BCA assay (Thermo Fisher Scientific). Samples were prepared for Western Blotting at 1 μg/μl, 10 μl loaded onto a 4%–15% SDS‐polyacrylamide gel and transferred to preactivated PDF membranes. Membranes were stained for Ponceau S, blocked with 5% nonfat milk powder, and incubated overnight at 4°C with the following primary antibodies at 1:2000 dilution: total OXPHOS rodent (Abcam Cat# ab110413, RRID:AB_2629281), PGC‐1α (GeneTex Cat# GTX37356, RRID:AB_11175466), NRF1 (GeneTex Cat# GTX103179, RRID:AB_11168915), TFAM (Abnova Corporation Cat# H00007019‐D01P, RRID:AB_1715621), Mfn2 (BioVision Cat# 3882–100, RRID:AB_2142625), Opa1 (Cell Signaling Technology Cat# 67589, RRID:AB_2799728), Fis1 (Abcam Cat# ab71498, RRID:AB_1271360), Drp1 (Novus Cat# NB110‐55288SS, RRID:AB_921147), Pink1 (Cell Signaling Technology Cat# 6946, RRID:AB_11179069), Parkin (Cell Signaling Technology Cat# 2132, RRID:AB_10693040), SOD1 (GeneTex Cat# GTX100554, RRID:AB_10618670), SOD2 (GeneTex Cat# GTX116093, RRID:AB_10624558), CAT (GeneTex Cat# GTX110704, RRID:AB_1949848), and GPX‐1 (GeneTex Cat# GTX116040, RRID:AB_2037097). Membranes were washed in TBST and incubated for 1 h with horseradish peroxidase‐conjugated anti‐rabbit IgG (Cell Signaling Technology Cat# 7074, RRID:AB_2099233) or anti‐mouse IgG (Cell Signaling Technology Cat# 7076, RRID:AB_330924) secondaries. Membranes were digitally imaged using a gel documentation system (Bio‐Rad). Raw target band densities were obtained and normalized by Ponceau values. Finally, band values were normalized by the mean of the YNG group to obtain percent change in comparison to the YNG group.

### 2.4 Oxidative damage markers

Lipid peroxidation and protein oxidation were assessed as markers of oxidative damage to both cortex and cerebellum. Lipid peroxidation levels were assessed by determining 4‐hydroxynonenal (4HNE) (Abcam Cat# ab46545, RRID:AB_722490) levels via Western Blotting as described above. Additionally, protein carbonyl levels were assessed using the Oxyblot protein oxidation detection kit (Millipore; #S7150) following manufacturer's instructions.

### 2.5 Citrate synthase and mitochondrial complexes activities

The activity of the enzyme citrate synthase and of the mitochondrial complexes I–III, II, and IV were determined spectrophotometrically at 37°C using cortex and cerebellum homogenates. Briefly, approximately 50 mg of each tissue were homogenized in a sucrose homogenization buffer using a glass dounce homogenizer according to Spinazzi et al. (2012). Samples were subjected to a series of freeze–thaw cycles before the assessment of enzymatic activities. Citrate synthase activity was determined by monitoring the increase in absorbance at 412 nm from the reduction of 5,5′‐dithiobis (2‐ nitrobenzoic acid) coupled to the reduction of Acetyl‐CoA (Trounce et al., 1996). The activity of the NADH cytochrome c oxireductase (complexes I‐III) was determined by the increase in absorbance at 550 nm after the addition of NADH (10 mM) (Spinazzi et al., 2012). Succinate dehydrogenase (complex II) activity assay was adapted from Spinazzi et al. (2012) and Trounce et al. (1996). In short, complex II activity was measured as a function of the decrease in absorbance at 600 nm from 2,6‐dichloroindophenol reduction in the presence of Antimycin A, KCN, and rotenone. Lastly, cytochrome c oxidase (complex IV) activity was measured by following the oxidation of reduced cytochrome c at 550 nm according to Spinazzi et al. (2012). The activity of the mitochondrial complexes was normalized by citrate synthase activity to account for changes in mitochondrial content and further normalized by the YNG group to be expressed as percent change from the YNG group.

### 2.6 Statistics

Normality of the dependent variables was assessed using Shapiro–Wilk tests. For normally distributed data, one‐way ANOVAs were utilized to test for differences between groups with Tukey's HSD post hoc tests when appropriate. Kruskal‐Wallis tests were used for variables that were not normally distributed. Welch One‐Way tests were used for normally distributed variables that presented heterogeneity of variances, followed by Games‐Howell tests when appropriate. Correlations between run distance and dependent variables were analyzed with Pearson or Spearman correlation tests. Statistical significance was established at *α* ≤ 0.050. Data are presented as mean ± standard deviation and ±95% confidence intervals (CI) are presented for statistically significant differences between groups. Statistics were performed using RStudio Version 2022.2.3.492 for Windows.

## 3 RESULTS

### 3.1 Running distance

Rats in the MA‐PA group ran on average 45.44 ± 25.49 km weekly and a total of 1579 ± 56 km throughout the entire duration of the study. There was no significant correlation between either average weekly or total distance run and any of the dependent variables (*p* > 0.05 for all correlations). A plot with the correlations for both cortex and cerebellum can be seen in Figure S1 (https://figshare.com/s/64114d9298ac4361ad27) and S2 (https://figshare.com/s/8ed6467d5643741d390e). A detailed description of the weekly running distance, as well as phenotype of the rats of the present study, including body weight, food intake, and weight of different tissues, can be found in Osburn et al. (2022).

### 3.2 Mitochondrial content

**FIGURE 1:**
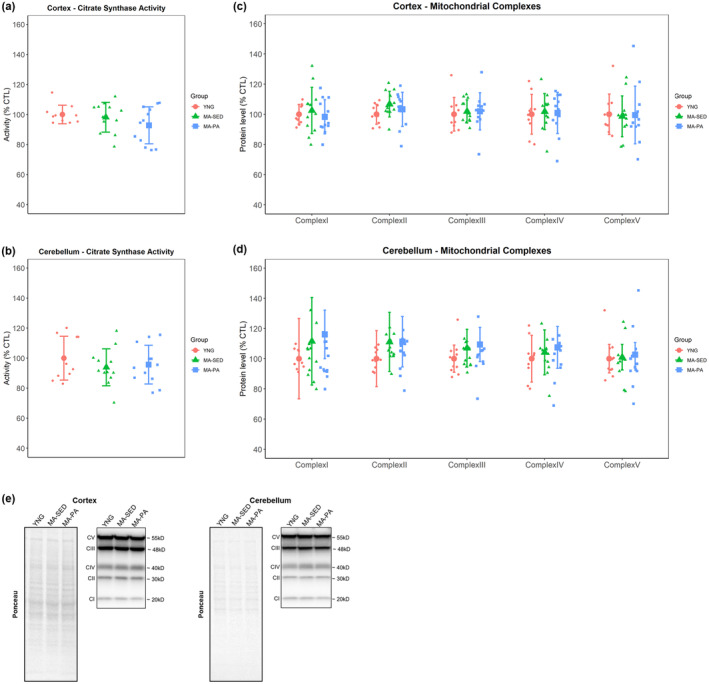
Markers of mitochondrial content. Citrate synthase activity (a, b), protein content of mitochondrial complexes I–V (c, d), and representative western blot images (e). No significant differences between groups were detected for markers of mitochondrial content in any of the tissues. Data are presented as mean ± SD and individual data points. YNG, young (*n* = 10); MA‐SED, middle‐aged sedentary (*n* = 12); MA‐PA, middle‐aged physical activity (*n* = 12).

There were no significant differences in citrate synthase activity between groups for either cortex (*p* = 0.245) or cerebellum (*p* = 0.558) (Figure 1a,b, respectively). No significant differences were detected for the protein levels of any of the mitochondrial complexes in either cortex; (CI: *p* = 0.690; CII: *p* = 0.240; CIII: *p* = 0.443; CIV: *p* = 0.949; CV: *p* = 0.948; Figure 1c) or cerebellum (CI: *p* = 0.436; CII: *p* = 0.101; CIII: *p* = 0.144; CIV: *p* = 0.502; CV: *p* = 0.754; Figure 1d).

### 3.3 Mitochondrial complexes activities

**FIGURE 2:**
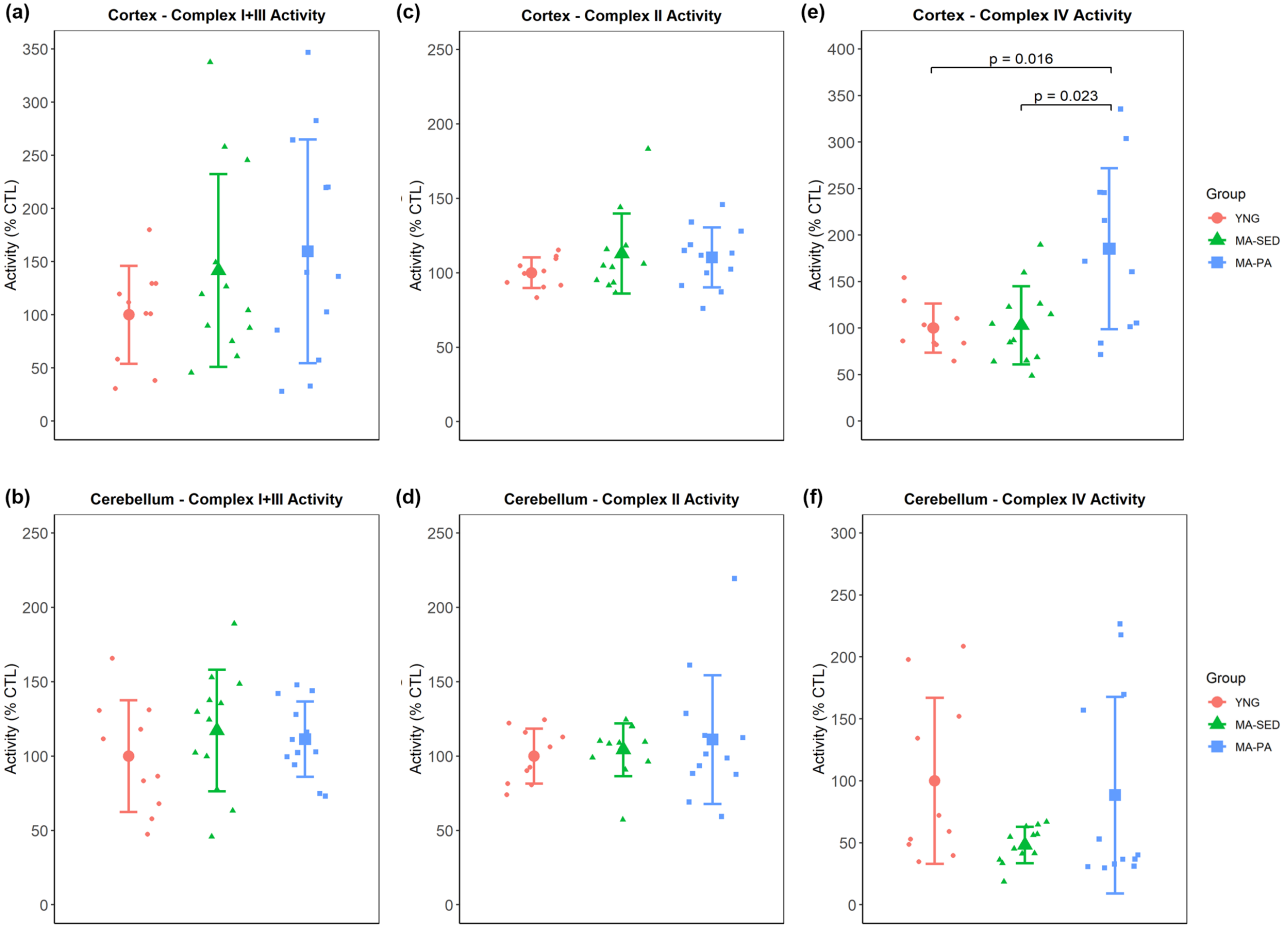
Enzymatic activity of mitochondrial complexes. Enzymatic activity of complex I + III (a, b), complex II (c, d), and complex IV (e, f). One‐way ANOVA revealed higher complex IV activity in MA‐PA compared to YNG and MA‐SED. Data are presented as mean ± SD and individual data points. YNG, young (*n* = 10); MA‐SED, middle‐aged sedentary (*n* = 12); MA‐PA, middle‐aged physical activity (*n* = 12).

No significant differences were detected for the activity of complex I + III (*p* = 0.275) or complex II (*p* = 0.334) in the cortex. However, complex IV activity was 85.3% (±95% CI [69.2%]) higher in the MA‐PA group compared to YNG (*p* = 0.016) and 82.30% (±95% CI [51.6%]) higher compared to MA‐SED (*p* = 0.023). In the cerebellum, no significant differences between groups were found for complex I + III (*p* = 0.520), complex II (*p* = 0.845), or complex IV (*p* = 0.284). Mitochondrial complexes activities for both cortex and cerebellum can be seen in Figure 2a–f.

### 3.4 Mitochondrial biogenesis

**FIGURE 3:**
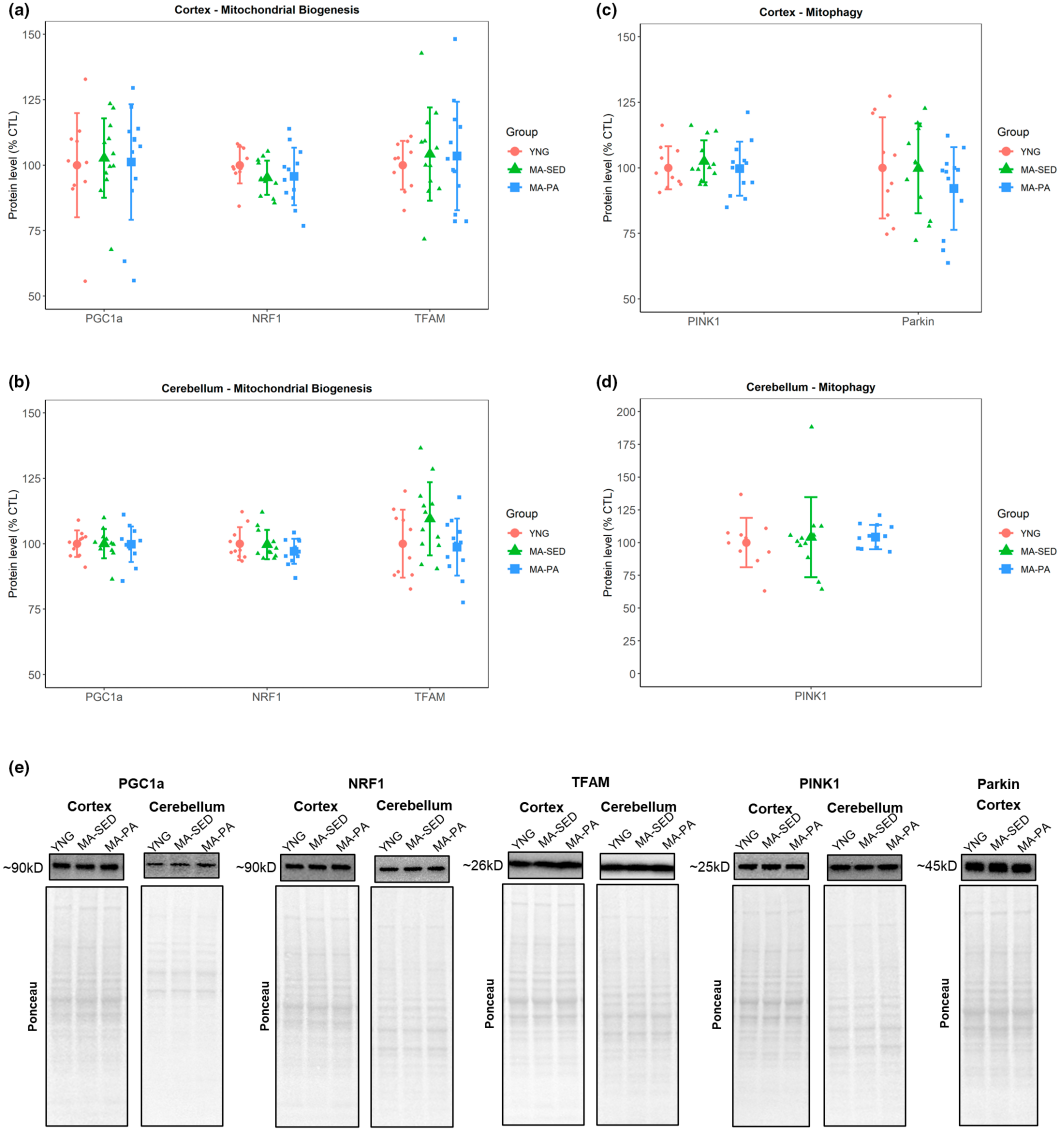
Markers of mitochondrial biogenesis and mitophagy. Markers of mitochondrial biogenesis (a, b) and mitophagy (c, d), with representative western blot images (e). No significant differences were detected for markers of mitochondrial biogenesis or mitophagy in the cortex and cerebellum. Data are presented as mean ± SD and individual data points. YNG, young (*n* = 10); MA‐SED, middle‐aged sedentary (*n* = 12); MA‐PA, middle‐aged physical activity (*n* = 12). Note that the following proteins share the same ponceau: Cortex: PGC‐1a and SOD2; TFAM and OPA1; Parkin, Mfn2, and Fis1; Cerebellum: PGC‐1a and SOD2; NRF1 and PINK1; TFAM and OPA1.

PGC‐1α, NRF‐1, and TFAM protein levels were analyzed as markers of mitochondrial biogenesis. There were no significant differences between groups in the levels of PGC‐1α (*p* = 0.754), NRF‐1 (*p* = 0.374), or TFAM (*p* = 0.834) in the cortex (Figure 3a). Similarly, no significant differences were detected in the cerebellum (PGC‐1α: *p* = 0.994; NRF‐1: *p* = 0.396; TFAM: *p* = 0.094; Figure 3b). TFAM levels in the cerebellum of the MA‐SED group showed a tendency to be higher than both YNG (9.50%, ±95% CI [13.35%]) and MA‐PA (10.82%, ±95% CI [12.73%]).

### 3.5 Mitophagy

PINK1 and Parkin were used as markers of mitophagy. There were no significant differences in the protein levels of PINK1 (*p* = 0.708) or Parkin (*p* = 0.408) in the cortex (Figure 3c). In the cerebellum, there was no significant difference between groups for PINK1 (*p* = 0.785; Figure 3d). Parkin was not detected in the cerebellum samples.

### 3.6 Mitochondrial fusion

**FIGURE 4:**
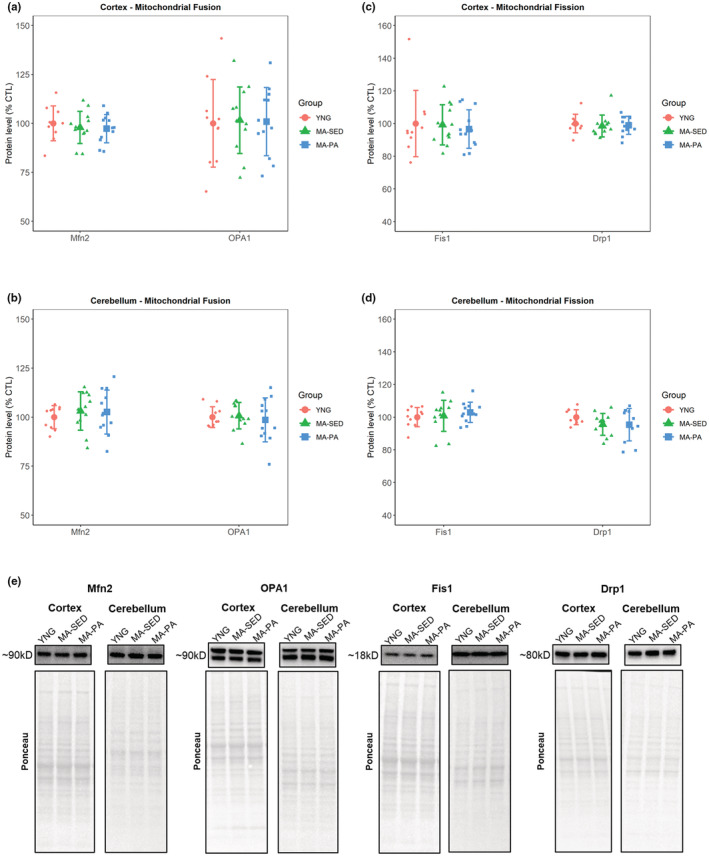
Markers of mitochondrial fusion and fission. Markers of mitochondrial fusion (a, b) and fission (c, d), with representative western blot images (e). No significant differences were detected for markers of mitochondrial fusion or fission in the cortex and cerebellum. Data are presented as mean ± SD and individual data points. YNG, young (*n* = 10); MA‐SED, middle‐aged sedentary (*n* = 12); MA‐PA, middle‐aged physical activity (*n* = 12). Note that the following proteins share the same ponceau: Cortex: Drp1, CAT, and GPX1; Cerebellum: Drp1, CAT, GPX1, and SOD1. Importantly, cerebellum GPX1 membrane was stripped after the image was captured and reincubated with SOD1.

There were no significant differences in the protein levels of markers of mitochondrial fusion in the cortex (Mfn2: *p* = 0.727; OPA1: *p* = 0.981; Figure 4a) or cerebellum (Mfn2: *p* = 0.718; OPA1: *p* = 0.865; Figure 4b). Long (L‐, top band) and short (S‐, bottom band) isoforms of OPA1 were further analyzed to identify any specific group‐differences. There were no significant differences in L‐OPA1 (*p* = 0.989) or S‐OPA1 (*p* = 0.961) in the cortex. Similarly, no differences were detected in L‐OPA1 (*p* = 0.854) or S‐OPA1 (*p* = 0.881) in the cerebellum.

### 3.7 Mitochondrial fission

The protein levels of markers of mitochondrial fission were not significantly different between groups in the cortex (Fis1: *p* = 0.962; Drp1: *p* = 0.471; Figure 4c) or cerebellum (Fis1: *p* = 0.643; Drp1: *p* = 0.299; Figure 4d).

### 3.8 Antioxidants

**FIGURE 5:**
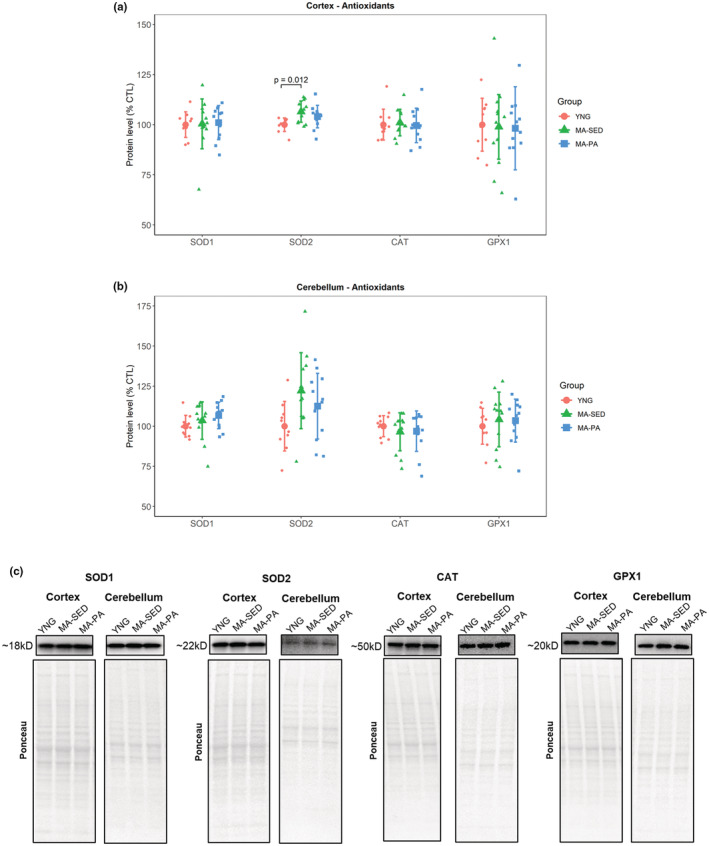
Endogenous antioxidant enzymes. Protein levels of antioxidants in the cortex (a) and cerebellum (b), with representative western blot images (c). The only significant difference detected was higher levels of SOD2 in the cortex of MA‐SED compared to YNG. Data are presented as mean ± SD and individual data points. YNG, young (*n* = 10); MA‐SED, middle‐aged sedentary (*n* = 12); MA‐PA, middle‐aged physical activity (*n* = 12). Note that proteins that share the same ponceau have been reported in previous images.

In the cortex, there were no significant differences between groups in the levels of SOD1 (*p* = 0.785), CAT (*p* = 0.904), and GPX1 (*p* = 0.429). However, SOD2 was 6.53% (±95% CI [5.23%], *p* = 0.012) higher in the MA‐SED group compared to YNG. In the cerebellum, no significant differences were detected for any of the antioxidants investigated (SOD1: *p* = 0.254; SOD2: *p* = 0.055; CAT: *p* = 0.920; GPX1: *p* = 0.771). SOD2 exhibited a tendency to be higher in the MA‐SED group compared to YNG (22.11%, ±95% CI [21.52%]). Protein levels of antioxidants can be seen in Figure 5a,b.

### 3.9 Oxidative damage

**FIGURE 6:**
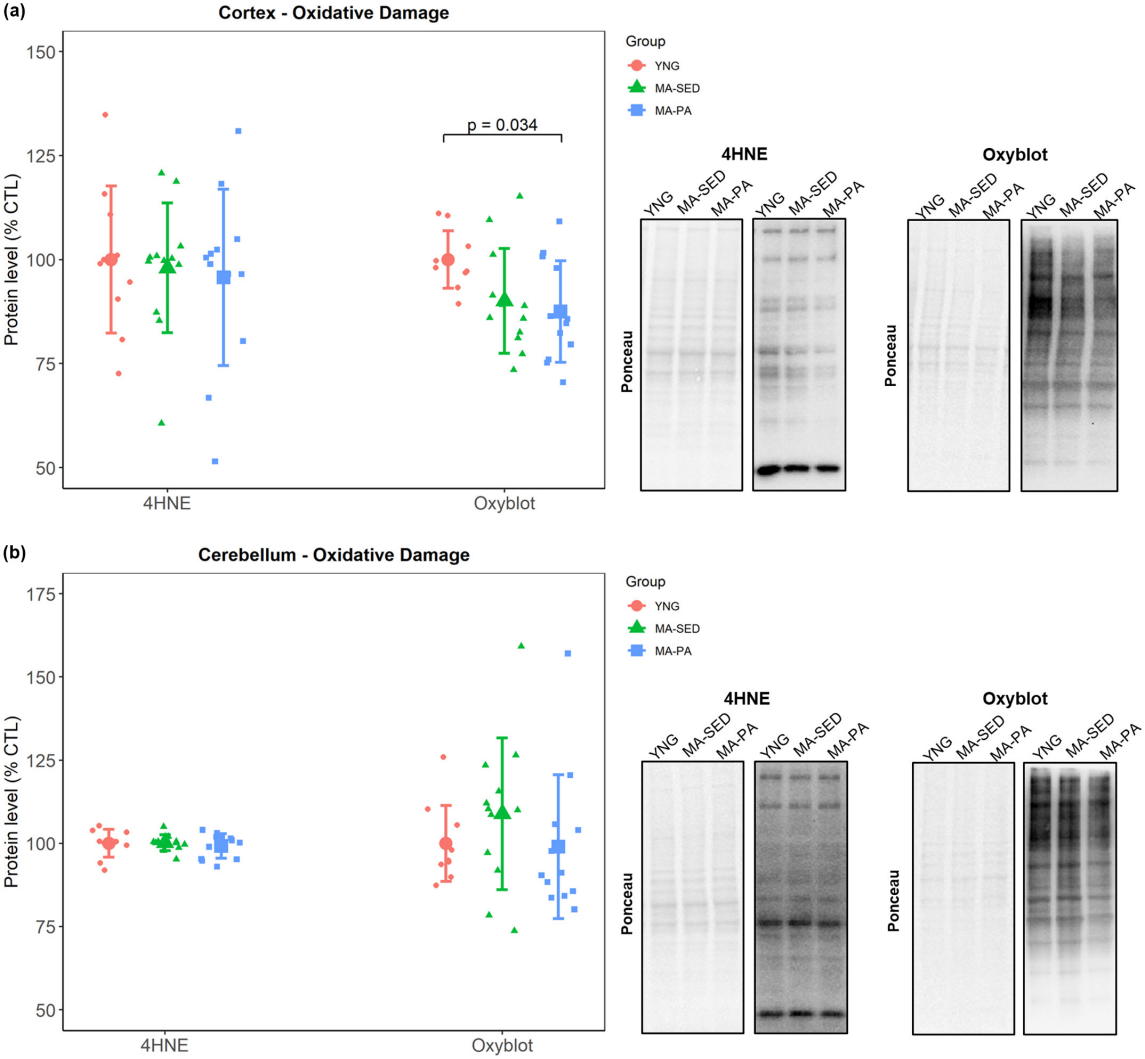
Markers of oxidative damage. Markers of oxidative damage in the cortex (a) and cerebellum (b), with representative western blot images. Levels of protein carbonyls were lower in the cortex of MA‐PA compared to YNG, while there were no significant differences in markers of oxidative damage in the cerebellum. Data are presented as mean ± SD and individual data points. YNG, young (*n* = 10); MA‐SED, middle‐aged sedentary (*n* = 12); MA‐PA, middle‐aged physical activity (*n* = 12).

In the cortex, there was no significant difference between groups in the levels of 4HNE (*p* = 0.864), while protein carbonyls were 12.49% (±95% CI [11.68%], *p* = 0.034) lower in the MA‐PA group compared to YNG (Figure 6a). No significant differences between groups were detected in 4HNE (*p* = 0.775) or protein carbonyls (*p* = 0.203) in the cerebellum (Figure 6b).

## 4 DISCUSSION

A disruption of mitochondrial function and the redox homeostasis of the cells have been considered underlying mechanisms in brain aging and in the development of several age‐related neurodegenerative diseases. Furthermore, substantial sex disparities have been shown for age‐related metabolic changes that occur in the brain, with females experiencing earlier declines in IGF signaling, glycolysis, mitochondrial function and remodeling, and redox balance (Zhao et al., 2016). Physical exercise has been considered an important therapeutic approach to counteract such age‐related changes in the brain and possibly prevent the development of neurodegenerative diseases (Bernardo et al., 2016; Marques‐Aleixo et al., 2012). In the current study, we investigated the effects of both aging and long‐term voluntary wheel running on mitochondrial physiology and redox state of the cortex and cerebellum of female rats. Overall, our results showed only minimal differences due to aging or voluntary wheel running in the variables interrogated.

While mitochondria are believed to have a major role in the aging process, the effects of aging on brain mitochondrial content are controversial. Our results showed no changes in citrate synthase activity or the protein content of the mitochondrial complexes in either cortex or cerebellum. These results are in agreement with previous studies showing no change in citrate synthase activity (Radak et al., 2001) or in the content of mitochondrial complexes I–V (Long et al., 2009) in the brains of male rats. Navarro et al. (2002), on the other hand, showed that while citrate synthase activity remained unchanged in the brains of middle‐aged (52 week‐old) female mice, this surrogate of mitochondrial content decreased in older (72 week‐old) mice, with a similar response in males, suggesting that mitochondrial content might only decrease at late stages of aging. Additionally, our results showed no significant effects of voluntary wheel running on mitochondrial content. Different studies have shown an increase in markers of mitochondrial content in response to exercise in different brain regions, including the cortex and cerebellum (Marques‐Aleixo et al., 2015; Steiner et al., 1985). However, it is important to note that both studies investigated the effects of short‐term exercise (8–12 weeks) in a sample of young male rodents, which may partially explain the discrepancies between studies. Even though it is possible that the beneficial effects of physical activity/exercise seen in young individuals are lost as they age, more studies investigating the effects of long‐term voluntary physical activity and/or controlled exercise on brain mitochondrial content are needed.

A decline in mitochondrial function has been considered one of the hallmarks of aging in several tissues (Lopez‐Otin et al., 2013), including the brain (Mattson & Arumugam, 2018). Several studies have revealed an age‐related decline in the activity of the electron transport system complexes in the brains of both male (Long et al., 2009; Navarro et al., 2004; Pollard et al., 2016) and female (Navarro et al., 2002, 2004) rodents. However, the effects of aging on mitochondrial respiration are less conclusive. Gusdon et al. (2017), for example, found that older mice exhibited decreased activity of complexes I + III but increased mitochondrial respiration driven by malate+glutamate and by succinate. In the current study, there were no significant effects of aging on the activity of complexes I + III, complex II, or complex IV in either cortex or cerebellum. Voluntary wheel running, however, did increase the activity of complex IV in the cortex. Exercise has been shown to have neuroprotective effects, enhancing cognitive function and delaying the development of neurodegenerative diseases (Marques‐Aleixo et al., 2012). Furthermore, exercise has been reported to increase the activity of mitochondrial complexes I (Marques‐Aleixo et al., 2015) and I + III (Gusdon et al., 2017) in the brains of both young and old rodents, respectively, while complex II activity was unaffected. Our results partially agree with the current literature, suggesting that complex II may be less responsive to exercise (Gusdon et al., 2017; Marques‐Aleixo et al., 2015; Navarro et al., 2004).

Besides the function of the electron transport system complexes, proper mitochondrial dynamics has been recognized as an important aspect of mitochondrial and cell health (Chen & Chan, 2009; Wai & Langer, 2016). The remodeling of the mitochondrial network depends on a fine‐tuned balance between mitochondrial biogenesis and mitophagy, and between mitochondrial fusion and fission. While mitochondrial biogenesis contributes to the increase, or the prevention of an age‐related decline, of mitochondrial content, mitophagy ensures that damaged portions of the mitochondrion are degraded, helping on the maintenance of a healthy mitochondrial population (Wang et al., 2021; Yoo & Jung, 2018). In alignment with the lack of changes in markers of mitochondrial content, we did not observe changes in markers of mitochondrial biogenesis or mitophagy with aging or voluntary wheel running. Marques‐Aleixo et al. (2015) and Steiner et al. (1985) found that short‐term exercise significantly increased markers of mitochondrial biogenesis in cortex but not in the cerebellum of young rodents. On the other hand, Gusdon et al. (2017) showed that 3 weeks of treadmill running did not change protein levels of PGC‐1α and TFAM in the cortex of young and old mice. However, to the best of our knowledge, there are no studies available that investigated the effects of long‐term voluntary physical activity and aging on brain mitochondrial biogenesis and/or mitophagy.

While a disruption of mitochondrial dynamics is implicated in the development of several neurodegenerative diseases (Chen & Chan, 2009; Gao et al., 2017), studies investigating changes in mitochondrial dynamics with healthy aging are scarce. In one of the few studies available, Thomsen et al. (2018) showed using both transmission electron microscopy and gene expression analysis, that aging led to increased fusion in the cortex, but increased fission in the hippocampus of middle‐aged male mice, highlighting that the changes are region‐specific. Our results showed no significant changes in markers of mitochondrial fusion or fission in the brain regions investigated with aging or physical activity. Twelve weeks of exercise has been shown to improve mitochondrial dynamics in the brains of young healthy (Marques‐Aleixo et al., 2015) and also in a Alzheimer's disease mouse model (Li et al., 2019; Yan et al., 2019). In older animals, Gusdon et al. (2017) found increased fission and no change in fusion markers in the cortex in response to 3 weeks of exercise. Regarding fusion, different OPA1 isoforms appear to have different roles, in which the long isoforms support mitochondrial fusion while the short isoforms appear to have a more energetic‐related role (Del Dotto et al., 2017). However, there were no isoform‐specific changes with either aging or physical activity. Again, no study to date has investigated the effects of long‐term physical activity/exercise on brain mitochondrial remodeling, which makes it difficult to draw assertive conclusions from our data.

Besides a decline in mitochondrial function, brain aging is accompanied by a disruption of the redox status. A decrease in the antioxidant defense system with a concomitant increase in oxidative damage is considered a strong driver of the aging process (Harman, 1956; Salmon et al., 2010; Sas et al., 2018). Several studies report an age‐related decline in enzymatic activity of various antioxidants (Bayliak et al., 2021; Navarro et al., 2004). Our results showed that overall, there was no change in the antioxidants investigated with either aging or voluntary wheel running. Even though it is possible that the aged rats herein were simply not old enough to exhibit the age‐related decrease in antioxidants commonly reported in the literature, the study of Bayliak et al. (2021) highlights that most changes (e.g., increased lipid peroxidation, decreased total antioxidant capacity and reduced glutathione) are already evident at middle age. Therefore, possible age‐related changes in the variables investigated herein should already be evident at MA. In addition, Meng et al. (2007) also found no change in the protein levels of SOD1, SOD2, CAT, and GPX with aging in the brains of rats. Therefore, it is possible that the age‐related decline in the antioxidant defense system comes from post‐translational modifications instead of changes in their content. In terms of oxidative damage markers, we did not observe significant changes in lipid peroxidation or protein oxidation with aging. However, long‐term voluntary wheel running decreased protein oxidation in the cortex. These results agree with Radak et al. (2001), who found that 9 weeks of swimming decreased protein carbonyls but not lipid peroxidation in the brain of middle‐aged rats.

Limitations of the present study include the fact that we did not have a group of male individuals to directly distinguish sex effects. In addition, we cannot rule out the possibility that changes in the variables investigated herein would be detected at later stages of aging or with more controlled exercise regimens.

## 5 CONCLUSIONS

In summary, the results of the current study showed minimal changes in several markers of mitochondrial content, function, and dynamics in the cortex and cerebellum of female rats in response to both aging and long‐term physical activity. Furthermore, the redox status of the tissues investigated remained overall unaltered. Our findings suggest that the brain mitochondrial physiology and redox homeostasis of females may be more resilient to the aging process than initially thought. In addition, the lack of effects of voluntary wheel running indicates that neurological benefits of exercise may be dependent on more controlled intensity and duration. It is important to note that most studies in the field used male rodents, making it difficult to compare the results of the current study and highlights the need for more studies using females. In addition, high variability in the literature with regards to rodents' strains, sex, length of aging, exercise regimens, and brain areas, makes it difficult to draw assertive conclusions about the subject.

## AUTHOR CONTRIBUTIONS

Paulo Mesquita: Conceptualization, formal analysis, investigation, writing – original draft, visualization; Shelby Osburn: Conceptualization, investigation, writing – review and editing; Josh Godwin: Investigation, writing – review and editing; Michael Roberts: Conceptualization, investigation, resources, supervision, funding acquisition, writing – review and editing; Andreas Kavazis: Conceptualization, resources, supervision, writing – review and editing.

## FUNDING INFORMATION

Funding for animals and assays was provided through a grant‐in‐aid (Florida A&M University) provided to M.D.R. The authors declare no conflicts of interest.

## CONFLICT OF INTEREST

None.

## Supporting information


Figure S1

Figure S2

## References

[phy215542-bib-0001] Bayliak, M. M. , Sorochynska, O. M. , Kuzniak, O. V. , Gospodaryov, D. V. , Demianchuk, O. I. , Vasylyk, Y. V. , Mosiichuk, N. M. , Storey, K. B. , Garaschuk, O. , & Lushchak, V. I. (2021). Middle age as a turning point in mouse cerebral cortex energy and redox metabolism: Modulation by every‐other‐day fasting. Experimental Gerontology, 145, 111182. 33290862 10.1016/j.exger.2020.111182

[phy215542-bib-0002] Bernardo, T. C. , Marques‐Aleixo, I. , Beleza, J. , Oliveira, P. J. , Ascensao, A. , & Magalhaes, J. (2016). Physical exercise and brain mitochondrial fitness: The possible role against Alzheimer's disease. Brain Pathology, 26, 648–663. 27328058 10.1111/bpa.12403 PMC8029062

[phy215542-bib-0003] Chen, H. , & Chan, D. C. (2009). Mitochondrial dynamics‐fusion, fission, movement, and mitophagy‐in neurodegenerative diseases. Human Molecular Genetics, 18, R169–R176. 19808793 10.1093/hmg/ddp326 PMC2758711

[phy215542-bib-0004] Del Dotto, V. , Mishra, P. , Vidoni, S. , Fogazza, M. , Maresca, A. , Caporali, L. , McCaffery, J. M. , Cappelletti, M. , Baruffini, E. , Lenaers, G. , Chan, D. , Rugolo, M. , Carelli, V. , & Zanna, C. (2017). OPA1 isoforms in the hierarchical organization of mitochondrial functions. Cell Reports, 19, 2557–2571. 28636943 10.1016/j.celrep.2017.05.073

[phy215542-bib-0005] Gao, J. , Wang, L. , Liu, J. , Xie, F. , Su, B. , & Wang, X. (2017). Abnormalities of mitochondrial dynamics in neurodegenerative diseases. Antioxidants (Basel), 6, 25. 28379197 10.3390/antiox6020025 PMC5488005

[phy215542-bib-0006] Grimm, A. , & Eckert, A. (2017). Brain aging and neurodegeneration: From a mitochondrial point of view. Journal of Neurochemistry, 143, 418–431. 28397282 10.1111/jnc.14037 PMC5724505

[phy215542-bib-0007] Gusdon, A. M. , Callio, J. , Distefano, G. , O'Doherty, R. M. , Goodpaster, B. H. , Coen, P. M. , & Chu, C. T. (2017). Exercise increases mitochondrial complex I activity and DRP1 expression in the brains of aged mice. Experimental Gerontology, 90, 1–13. 28108329 10.1016/j.exger.2017.01.013 PMC5346470

[phy215542-bib-0008] Harman, D. (1956). Aging: A theory based on free radical and radiation chemistry. Journal of Gerontology, 11, 298–300. 13332224 10.1093/geronj/11.3.298

[phy215542-bib-0009] Li, B. , Liang, F. , Ding, X. , Yan, Q. , Zhao, Y. , Zhang, X. , Bai, Y. , Huang, T. , & Xu, B. (2019). Interval and continuous exercise overcome memory deficits related to beta‐amyloid accumulation through modulating mitochondrial dynamics. Behavioural Brain Research, 376, 112171. 31445975 10.1016/j.bbr.2019.112171

[phy215542-bib-0010] Long, J. , Gao, F. , Tong, L. , Cotman, C. W. , Ames, B. N. , & Liu, J. (2009). Mitochondrial decay in the brains of old rats: Ameliorating effect of alpha‐lipoic acid and acetyl‐L‐carnitine. Neurochemical Research, 34, 755–763. 18846423 10.1007/s11064-008-9850-2 PMC2790461

[phy215542-bib-0011] Lopez‐Otin, C. , Blasco, M. A. , Partridge, L. , Serrano, M. , & Kroemer, G. (2013). The hallmarks of aging. Cell, 153, 1194–1217. 23746838 10.1016/j.cell.2013.05.039 PMC3836174

[phy215542-bib-0012] Lores‐Arnaiz, S. , & Bustamante, J. (2011). Age‐related alterations in mitochondrial physiological parameters and nitric oxide production in synaptic and non‐synaptic brain cortex mitochondria. Neuroscience, 188, 117–124. 21600964 10.1016/j.neuroscience.2011.04.060

[phy215542-bib-0013] Lores‐Arnaiz, S. , Lombardi, P. , Karadayian, A. G. , Orgambide, F. , Cicerchia, D. , & Bustamante, J. (2016). Brain cortex mitochondrial bioenergetics in synaptosomes and non‐synaptic mitochondria during aging. Neurochemical Research, 41, 353–363. 26818758 10.1007/s11064-015-1817-5

[phy215542-bib-0014] Marques‐Aleixo, I. , Oliveira, P. J. , Moreira, P. I. , Magalhaes, J. , & Ascensao, A. (2012). Physical exercise as a possible strategy for brain protection: Evidence from mitochondrial‐mediated mechanisms. Progress in Neurobiology, 99, 149–162. 22940590 10.1016/j.pneurobio.2012.08.002

[phy215542-bib-0015] Marques‐Aleixo, I. , Santos‐Alves, E. , Balca, M. M. , Rizo‐Roca, D. , Moreira, P. I. , Oliveira, P. J. , Magalhaes, J. , & Ascensao, A. (2015). Physical exercise improves brain cortex and cerebellum mitochondrial bioenergetics and alters apoptotic, dynamic and auto(Mito)phagy markers. Neuroscience, 301, 480–495. 26116519 10.1016/j.neuroscience.2015.06.027

[phy215542-bib-0016] Mattson, M. P. , & Arumugam, T. V. (2018). Hallmarks of brain aging: Adaptive and pathological modification by metabolic states. Cell Metabolism, 27, 1176–1199. 29874566 10.1016/j.cmet.2018.05.011 PMC6039826

[phy215542-bib-0017] Meng, Q. , Wong, Y. T. , Chen, J. , & Ruan, R. (2007). Age‐related changes in mitochondrial function and antioxidative enzyme activity in fischer 344 rats. Mechanisms of Ageing and Development, 128, 286–292. 17270247 10.1016/j.mad.2006.12.008

[phy215542-bib-0018] Navarro, A. , Gomez, C. , Lopez‐Cepero, J. M. , & Boveris, A. (2004). Beneficial effects of moderate exercise on mice aging: Survival, behavior, oxidative stress, and mitochondrial electron transfer. American Journal of Physiology. Regulatory, Integrative and Comparative Physiology, 286, R505–R511. 14615275 10.1152/ajpregu.00208.2003

[phy215542-bib-0019] Navarro, A. , Sanchez Del Pino, M. J. , Gomez, C. , Peralta, J. L. , & Boveris, A. (2002). Behavioral dysfunction, brain oxidative stress, and impaired mitochondrial electron transfer in aging mice. American Journal of Physiology. Regulatory, Integrative and Comparative Physiology, 282, R985–R992. 11893601 10.1152/ajpregu.00537.2001

[phy215542-bib-0020] Osburn, S. C. , Mesquita, P. , Neal, F. K. , Rumbley, M. , Holmes, M. T. , Ruple, B. A. , Mobley, C. B. , Brown, M. D. , McCullough, D. J. , Kavazis, A. N. , & Roberts, M. D. (2022). Long‐term voluntary wheel running effects on markers of Long interspersed nuclear Element‐1 in skeletal muscle, liver, and brain tissue of female rats. American Journal of Physiology. Cell Physiology, 323, C907–C919. 35938680 10.1152/ajpcell.00234.2022

[phy215542-bib-0021] Pollard, A. K. , Craig, E. L. , & Chakrabarti, L. (2016). Mitochondrial complex 1 activity measured by spectrophotometry is reduced across all brain regions in ageing and more specifically in neurodegeneration. PLoS One, 11, e0157405. 27333203 10.1371/journal.pone.0157405 PMC4917223

[phy215542-bib-0022] Radak, Z. , Kaneko, T. , Tahara, S. , Nakamoto, H. , Pucsok, J. , Sasvari, M. , Nyakas, C. , & Goto, S. (2001). Regular exercise improves cognitive function and decreases oxidative damage in rat brain. Neurochemistry International, 38, 17–23. 10913684 10.1016/s0197-0186(00)00063-2

[phy215542-bib-0023] Salmon, A. B. , Richardson, A. , & Perez, V. I. (2010). Update on the oxidative stress theory of aging: Does oxidative stress play a role in aging or healthy aging? Free Radical Biology & Medicine, 48, 642–655. 20036736 10.1016/j.freeradbiomed.2009.12.015 PMC2819595

[phy215542-bib-0024] Sas, K. , Szabo, E. , & Vecsei, L. (2018). Mitochondria, oxidative stress and the kynurenine system, with a focus on ageing and neuroprotection. Molecules, 23, 191. 29342113 10.3390/molecules23010191 PMC6017505

[phy215542-bib-0025] Spinazzi, M. , Casarin, A. , Pertegato, V. , Salviati, L. , & Angelini, C. (2012). Assessment of mitochondrial respiratory chain enzymatic activities on tissues and cultured cells. Nature Protocols, 7, 1235–1246. 22653162 10.1038/nprot.2012.058

[phy215542-bib-0026] Steiner, J. L. , Murphy, E. A. , McClellan, J. L. , Carmichael, M. D. , & Davis, J. M. (1985). Exercise training increases mitochondrial biogenesis in the brain. Journal of Applied Physiology, 111(1066–1071), 2011. 10.1152/japplphysiol.00343.2011 21817111

[phy215542-bib-0027] Thomsen, K. , Yokota, T. , Hasan‐Olive, M. M. , Sherazi, N. , Fakouri, N. B. , Desler, C. , Regnell, C. E. , Larsen, S. , Rasmussen, L. J. , Dela, F. , Bergersen, L. H. , & Lauritzen, M. (2018). Initial brain aging: Heterogeneity of mitochondrial size is associated with decline in complex I‐linked respiration in cortex and hippocampus. Neurobiology of Aging, 61, 215–224. 29031832 10.1016/j.neurobiolaging.2017.08.004

[phy215542-bib-0028] Trounce, I. A. , Kim, Y. L. , Jun, A. S. , & Wallace, D. C. (1996). Assessment of mitochondrial oxidative phosphorylation in patient muscle biopsies, lymphoblasts, and transmitochondrial cell lines. Methods in Enzymology, 264, 484–509. 8965721 10.1016/s0076-6879(96)64044-0

[phy215542-bib-0029] Wai, T. , & Langer, T. (2016). Mitochondrial dynamics and metabolic regulation. Trends in Endocrinology and Metabolism, 27, 105–117. 26754340 10.1016/j.tem.2015.12.001

[phy215542-bib-0030] Wang, X. L. , Feng, S. T. , Wang, Z. Z. , Chen, N. H. , & Zhang, Y. (2021). Role of mitophagy in mitochondrial quality control: Mechanisms and potential implications for neurodegenerative diseases. Pharmacological Research, 165, 105433. 33454337 10.1016/j.phrs.2021.105433

[phy215542-bib-0031] Yan, Q. W. , Zhao, N. , Xia, J. , Li, B. X. , & Yin, L. Y. (2019). Effects of treadmill exercise on mitochondrial fusion and fission in the hippocampus of APP/PS1 mice. Neuroscience Letters, 701, 84–91. 30796962 10.1016/j.neulet.2019.02.030

[phy215542-bib-0032] Yoo, S. M. , & Jung, Y. K. (2018). A molecular approach to Mitophagy and mitochondrial dynamics. Molecules and Cells, 41, 18–26. 29370689 10.14348/molcells.2018.2277 PMC5792708

[phy215542-bib-0033] Zhao, L. , Mao, Z. , Woody, S. K. , & Brinton, R. D. (2016). Sex differences in metabolic aging of the brain: Insights into female susceptibility to Alzheimer's disease. Neurobiol Aging, 42, 69–79. 27143423 10.1016/j.neurobiolaging.2016.02.011 PMC5644989

[phy215542-bib-0034] Zhu, D. , Montagne, A. , & Zhao, Z. (2021). Alzheimer's pathogenic mechanisms and underlying sex difference. Cellular and Molecular Life Sciences, 78, 4907–4920. 33844047 10.1007/s00018-021-03830-w PMC8720296

