# Supplementary figures and images for: Effects of aging and long‐term physical activity on mitochondrial physiology and redox state of the cortex and cerebellum of female rats
Source: Physiol Rep. 2022 Dec 21;10(24):e15542. doi: 10.14814/phy2.15542 (PMC9771693; doi:10.14814/phy2.15542)

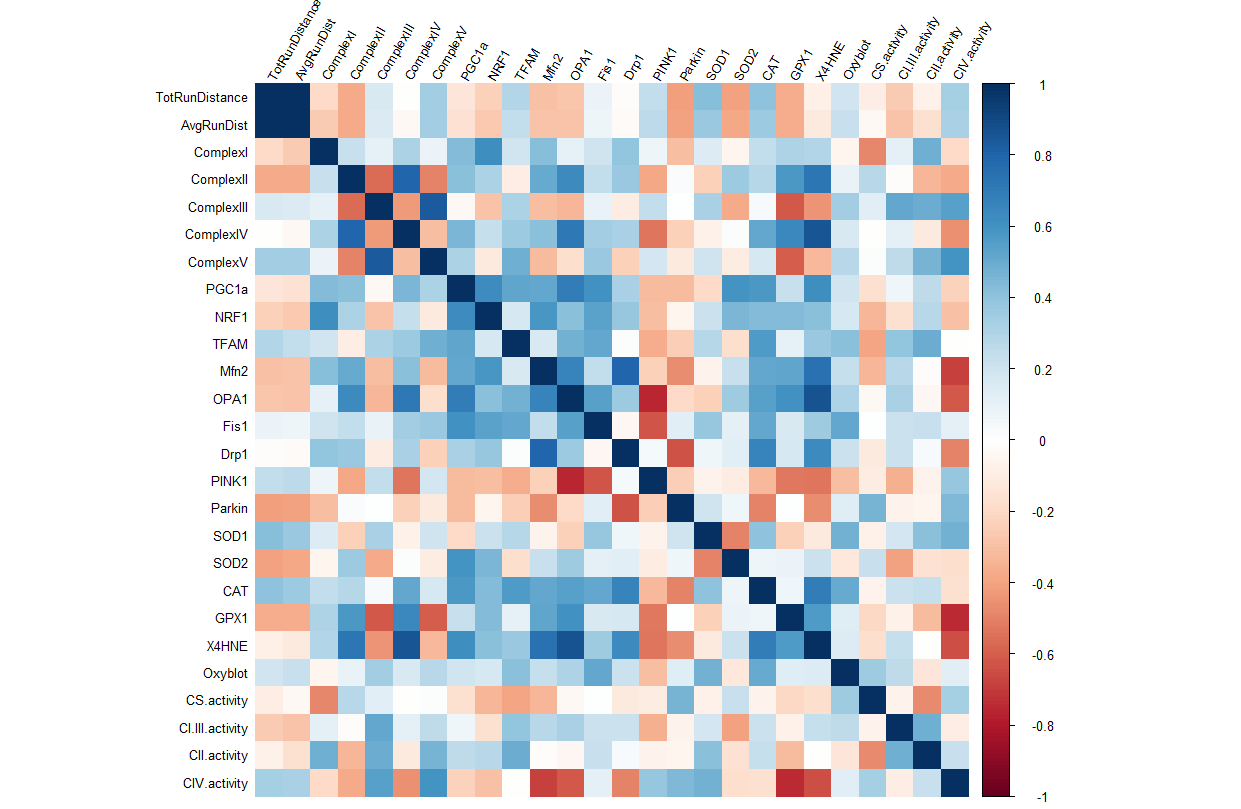

Supplement: Supplementary file 1 — Figure S1 [file PHY2-10-e15542-s001.tiff]

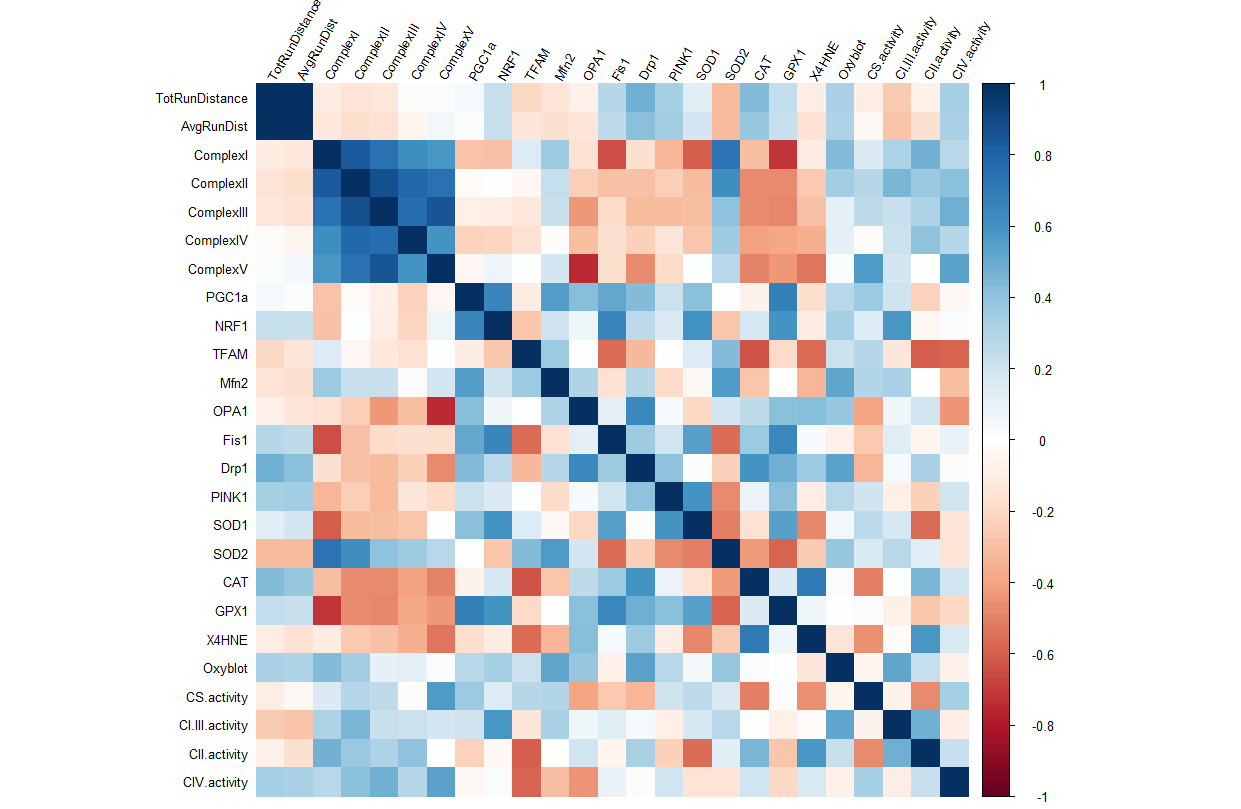

Supplement: Supplementary file 2 — Figure S2 [file PHY2-10-e15542-s002.tiff]
